# Primary cardiac dedifferentiated liposarcoma in a middle-aged female: a case report

**DOI:** 10.1186/s13019-019-0973-0

**Published:** 2019-08-30

**Authors:** Jiayu Shen, Zhi Fang, Yahan Zhang, Yingqiang Guo

**Affiliations:** 1 Department of Cardiovascular Surgery, West China Hospital, Sichuan University, No.37 Guo Xue Alley, Chengdu, Sichuan People’s Republic of China 61004; 2 Department of Pathology, West China Hospital, Sichuan University, No.37 Guo Xue Alley, Chengdu, 610041 China

**Keywords:** Dedifferentiated liposarcoma, Heart failure, Intracavitary atrial mass, Pleural effusion, Primary malignant cardiac tumor

## Abstract

**Background:**

Primary malignant cardiac tumors are extremely rare and can present with the same nonspecific characteristics as benign primary cardiac tumors. We herein describe a middle-aged female with an intracavitary, irregular atrial mass who experienced partial surgical resection. The atrial mass which was recognized as myxoma before surgery was finally diagnosed as dedifferentiated liposarcoma (DDLPS) by postoperative pathological examination.

**Case presentation:**

The patient, a 61-year-old female, presented to the emergency room because of progressive chest congestion and shortage of liberties for 6 months and orthopnoea and paroxysmal nocturnal dyspnea for 3 days. The laboratory examinations confirmed no abnormalities. The thoracic computed tomography (CT) scan showed massive hydropericardium, pleural effusion and left atrium occupying lesion. The transesophageal echocardiography (TEE) confirmed an intracavitary and irregular left atrial mass, limiting the mitral valve inflow and pulmonary venous blood reflux. The positron emission tomography/computed tomography (PET/CT) revealed high grade fluorodeoxyglucose uptake only in the intracavitary mass which near the mitral valve. According to operative exploration, the intracavitary mass had invaded the mitral annulus and posterior wall of left ventricle which cannot be resected completely, we did merely partial surgical resection to relieve the patient’s symptoms. Postoperative immunohistochemical stain confirmed the diagnosis of DDLPS. The patient was transferred to the oncology department for further therapy. Unfortunately, the patient was detected with brain metastasis 1 month later and died within 5 months after the surgery.

**Conclusions:**

Primary cardiac DDLPS is an extremely rare histological subtype of undifferentiated pleomorphic sarcomas which present the same nonspecific characteristics as benign primary cardiac tumors. Even though surgical resection combined with chemotherapy or radiotherapy remains the mainstream treatment strategy, the prognosis of cardiac malignancy is poor with high mortality. Novel management strategies need to be further explored.

**Electronic supplementary material:**

The online version of this article (10.1186/s13019-019-0973-0) contains supplementary material, which is available to authorized users.

## Background

The presence of a heart tumor was first identified in 1559. However, it was not until 1934 that the first clinical diagnosis of a primary heart sarcoma was reported [1]. The autopsy incidence of the primary cardiac neoplasm is extremely low (0.0001–0.030%) or around 1 in every 500 cardiovascular surgical cases [2, 3]. The benign cardiac tumors such as myxoma account for 75%. Of the remaining 25% of tumors that are identified as being malignant, cardiac sarcomas comprise 95% of cases [4]. The clinical characteristics of the malignant primary cardiac tumors cannot be significantly distinguished from benign primary cardiac tumors. We herein describe a middle-aged female with an intracavitary, irregular atrial mass experienced partial surgical resection. The atrial mass which was suspected as myxoma before surgery was finally diagnosed as DDLPS by postoperative pathological examination.

## Case presentation

**Fig. 1:**
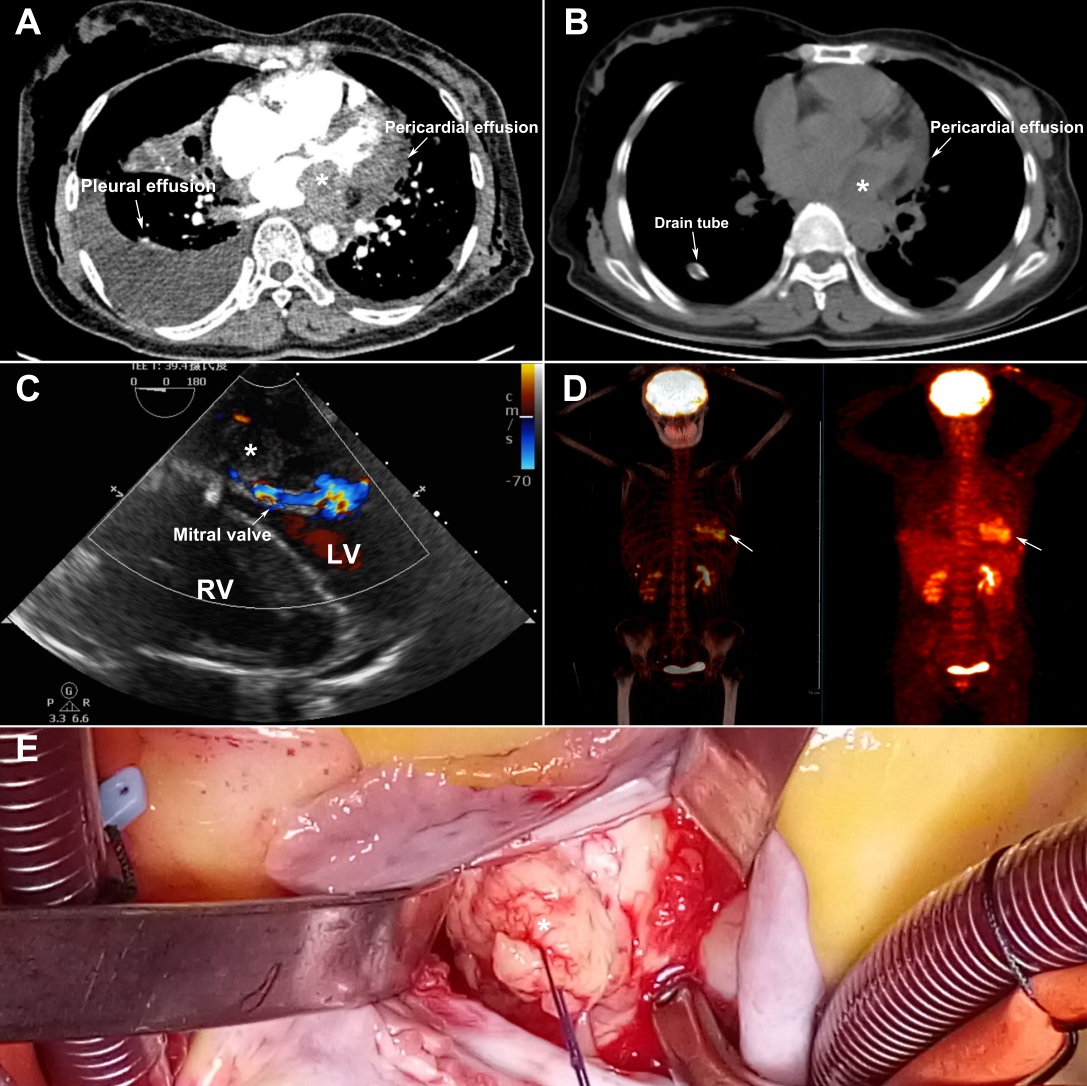
Preoperative examinations and intraoperative findings: **a** CT scans revealed occupying lesion in left atrium and massive hydropericardium and pleural effusion (asterisk and arrows); **b** The massive right pleural effusion reduced significantly after chest drainage; **c** TEE showed the left atrial mass limited the mitral valve inflow significantly; **d** PET/CT confirmed high grade fluorodeoxyglucose uptake only in the intracavitary mass (arrows); **e** Operative exploration confirmed the atrial mass had invaded the mitral annulus and posterior wall of left ventricle. * The left atrial mass; RV, right ventricle; LV, left ventricle; TEE, Transesophageal echocardiography; PET/CT, Positron emission tomography/computed tomography


A 61-year-old female presented to the emergency room with a 6-month history of progressive chest congestion and shortage of liberties with no abnormal medical history. During the last 3 days, the patient experienced orthopnoea and paroxysmal nocturnal dyspnea. On admission, physical examination revealed body temperature of 37.6 °C, heart rate 120 beats/min, respiratory rate 35 per minute, blood pressure 122/75 mmHg and right lung respiratory sound was reduced. Laboratory examinations demonstrated mild leukocytosis (leukocyte count 10.5 × 10^9^/L). Thoracic CT scan revealed occupying lesion in left atrium, accompanied with massive hydropericardium and pleural effusion. (Fig. 1a) Simultaneously right pleural puncture and chest drainage was done to relieve the patient’s symptoms. (Fig. 1b) Transesophageal echocardiography (TEE) showed an irregular left atrial mass (measuring 5.0*5.2 cm), limiting the mitral valve inflow and covering the ostia of the left-side pulmonary veins. (Fig. 1c; Additional file 1) The patient was referred for a PET/CT for further evaluation. Maximum intensity projection images revealed high grade fluorodeoxyglucose uptake only in the intracavitary mass which near the mitral valve. (Fig. 1d).

**Additional file 1:** The video of preoperative TEE. (MP4 15574 kb)


The patient received emergency surgical intervention under cardiopulmonary bypass since her clinical status deteriorated quickly. The pericardium effusion was drained firstly after pericardiotomy. Operative exploration confirmed complete resection could not be achieved, since the broad intracavitary mass surrounded with fibrotic density had invaded the mitral annulus and posterior wall of left ventricle (Fig. 1e). The intraoperative impression of the frozen section revealed that the lesion was predominantly composed of hyperchromatic spindled cells, and was highly suspicious for spindle cell malignancy. Based on these results, we did merely partial surgical resection only for the protrusive part of the intracavitary mass to minimize the restriction to the pulmonary venous blood reflux and mitral valve flow as much as possible.

**Fig. 2:**
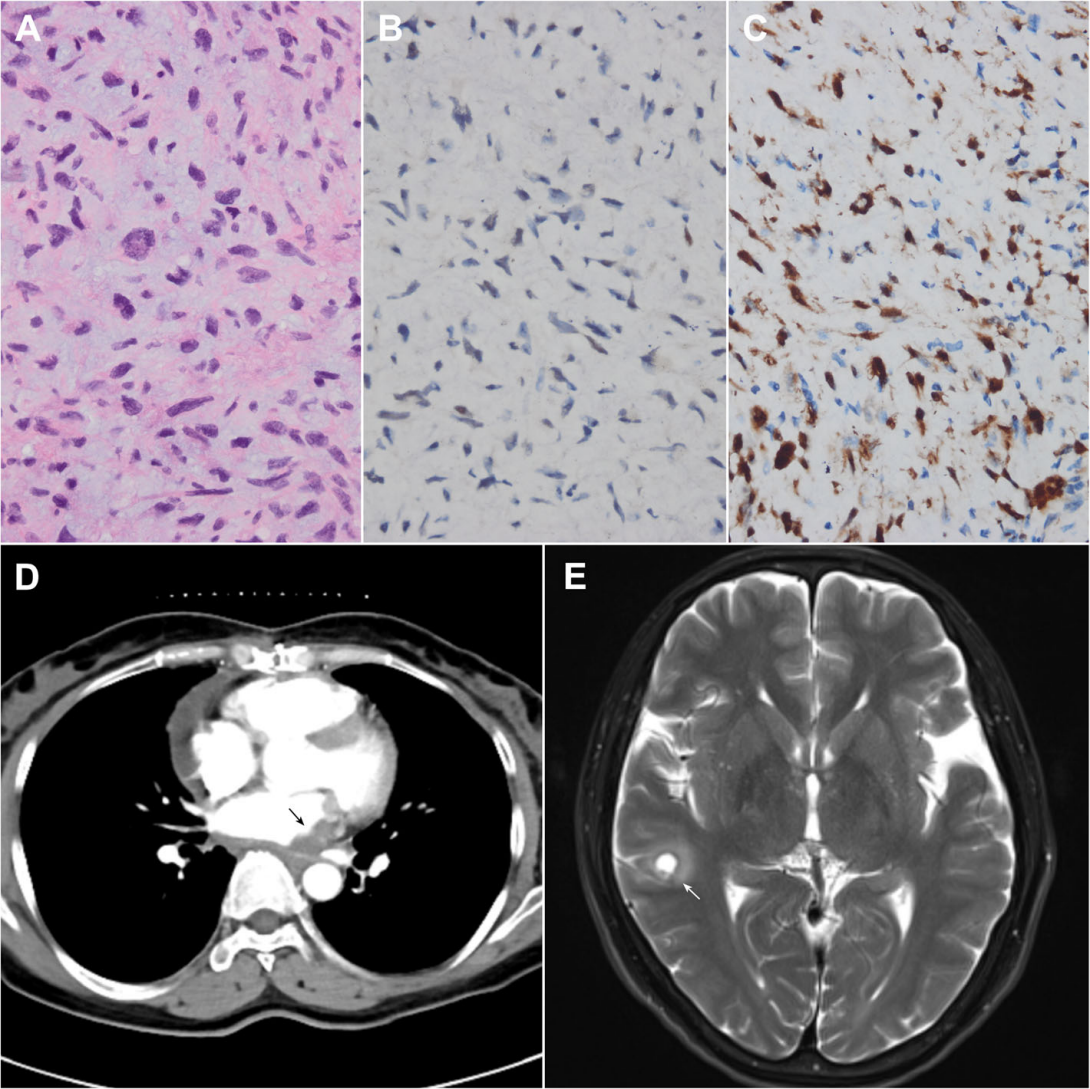
Pathological findings and follow-up imaging examinations: **a** Markedly atypical cells which lack specific morphological features of differentiation was found by hematoxylin–eosin staining (H&E, 400×); **b** and **c** Immunohistochemical staining of the intracavitary mass is strongly positive for MDM2 and CDK4 (Immunostaining, 400×); **d** Postoperative thoracic CT scan confirmed the mitral obstruction was totally relieved after partial surgical resection (black arrow); **e** Postoperative brain MRI examination showed the brain metastasis (white arrow)

After the surgery, the removed left atrium mass was sent for further histopathological examination. Hematoxylin–eosin staining showed the mass was composed of markedly atypical cells which lack specific morphological features of differentiation (Fig. 2a). Immunohistochemical stain revealed malignant cells strongly positive for MDM2 and CDK4 (Fig. 2b and c), supporting the diagnosis of DDLPS. Postoperative CT scan confirmed the mitral obstruction was totally relieved. (Fig. 2d) The patient was then transferred to the oncology department for further molecular targeting therapy. Unfortunately, the patient was detected with brain metastasis 1 month later (Fig. 2e) and died within 5 months after the surgery.

## Discussion and conclusions

DDLPS is one of the most common types of soft tissue sarcoma which represents over 60% of all liposarcoma [5] and is almost universally associated with amplification of chromosome segment 12q13–15, which carries the oncogenes MDM2 and CDK4 [6–8]. DDLPS usually originates from adipogenic precursor cells in deep soft tissue, such as that inside the thigh or in the retroperitoneum and frequently presents as a large (> 15 cm), painless, retroperitoneal mass [9]. Being a non-lipotomatous, highly cellular malignancy with a much more aggressive phenotype, DDLPS has a high rate of local recurrence and systemic metastases [10].

In primary cardiac DDLPS, no sex predilection is apparent. Although cases have been identified among a wide range of ages, most patients that are affected are younger than 45 years of age [11, 12]. The clinical characteristics of the primary cardiac DDLPS mainly depend on the location and infiltration and has no specific characteristics from benign primary cardiac tumors. A study involving 34 patients with primary malignant cardiac tumors observed over a 32-year period reported symptoms such as dyspnoea on exertion (79%), chest pain (38%), cough (21%), paroxysmal nocturnal dyspnea (12%), haemoptysis (12%), embolic events (9%), fever (9%), syncope (6%), and orthopnoea (6%) [3, 13]. In this case, the intracavitary left atrial mass limited the mitral valve flow and pulmonary venous blood reflux, causing paroxysmal nocturnal dyspnea, orthopnoea and polyserous effusions which are symptoms of left heart failure.

The DDLPS in cardiac chambers often appear as large, irregular, low-attenuation lesions on multidetector CT, and can be either noninvasive/focal or demonstrate infiltration into the myocardium [14, 15]. As we can observe from the preoperative CT scan, the intracavitary mass has integrated with the myocardium, suggesting the myocardial invasion of the mass. TEE can provide us with more information about the contiguity of the intracavitary mass and other cardiac structures. We also did a further PET/CT for our patient to determine that the intracavitary left atrial mass originates from the myocardium. MRI can also serve as a reference. Like other sarcomas, DDLPS generally appear isointense on T1-weighted images and hyperintense on T2-weighted images, with a heterogenous, delayed-enhancement pattern postcontrast that is related to the underlying composition of the intracavitary mass [16]. Moreover, whole body MRI is a useful technique in detecting distant metastases. However, in this case, the deterioration our patient’s clinical status seemed to suddenly accelerated after PET-CT and the unstable hemodynamics prevented us from performing other time-consuming examinations. Unlike angiosarcomas, which seem to have a predilection for the right atrium, primary cardiac DDLPS are mainly left-sided and are not likely to be performed with invasive biopsy. Meanwhile, given that the rarity of primary cardiac DDLPS, data that allow reliable and noninvasive diagnosis are limited and the definitive inferences about optimal therapy remains to be explored.

Even though cardiac tumors present a particular challenge for cardiac surgeons, surgical resection should be attempted in the setting of localized disease, provided the patient has acceptable performance status, in that it seems to provide the best option for palliative care and potential cure [4, 17]. Complete surgical resection combined with adjuvant chemotherapy and/or local radiotherapy is mandatory to reduce the risk of local recurrence and distant metastasis [4, 13]. However, the anatomical location and the range of infiltration of the tumor put strain on the acceptance of complete resection as the main treatment modality and it is also worth to mentioning that heart failure is commonly seen in the early course of the left-sided malignant cardiac tumor, and neoadjuvant chemotherapy is usually contraindicated prior to surgery [18]. Implantation of an artificial heart and cardiac transplantation represent an emerging and promising treatment strategy for young patients with no evidence of distant metastasis and isolated unresectable cardiac involvement in recent years [19]. If the hemodynamics of the patient with unresectable tumors remain stable and the donor heart can be obtained in time, heart transplantation can be considered as primary treatment option. However, for patients with initial hemodynamic instability, emergency surgical resection should be done as soon as possible, because this condition can deteriorate in a short time. In this case, as the patient with rapidly deteriorated left heart function, we did emergency partial surgical resection to relieve the restriction on mitral valve flow and pulmonary venous blood reflux, leaving the patient in the danger of neoplasm recurrence and metastasis. Even though the patient received regular chemotherapy immediately after surgery, brain metastasis was detected in the early phase and the prognosis was poor.

With a better understanding of the molecular and genetic factors promoting the growth of tumors, more specific and patient-targeted agents such as palbociclib [20] (a cyclin-dependent kinase 4/6 inhibitor used in the treatment of liposarcomas) and olaratumab [21] (a monoclonal antibody directed against platelet-derived growth factor-α for undifferentiated pleomorphic sarcoma and liposarcoma, in combination with doxorubicin) which have been used for certain noncardiac sarcomas may have a role in the treatment of cardiac sarcomas.

In conclusion, primary cardiac DDLPS is an extremely rare histological subtype of undifferentiated pleomorphic sarcomas which present the same nonspecific characteristics as benign primary cardiac tumors. Even though surgical resection combined with chemotherapy or radiotherapy remains the mainstream treatment strategy, the prognosis of cardiac malignancy is still poor with high mortality. Novel management strategies such as molecular targeting therapy in the treatment of cardiac sarcomas needs to be further explored.

## Data Availability

All data generated or analyzed during this study are included in this published article.

## Abbreviations

CT: Computed tomography
DDLPS: Dedifferentiated liposarcoma
PET/CT: Positron emission tomography/computed tomography
